# The relationship between rural information consumption and regional economic development with spatial Durbin model—A case study of Zhejiang, China

**DOI:** 10.1371/journal.pone.0281405

**Published:** 2023-02-13

**Authors:** Haidong Zhong, Jinhui Zhang, Wenlan Song

**Affiliations:** 1 School of Economics and Management, Ningbo University of Technology, Ningbo, Zhejiang, China; 2 School of Logistics and E-commerce, Zhejiang Wanli University, Ningbo, Zhejiang, China; Shenzhen University, CHINA

## Abstract

Information consumption is a kind of economic activity that directly or indirectly takes information products and information services as consumption objects. Most of the existing studies have established the "formula” of rural information consumption in promoting economic and social development, while few efforts have been devoted to study the quantitative reciprocities between them. Therefore, this study aims to investigate the relationship between information consumption in rural areas and regional economic development with a case study of Zhejiang, China. Based on rural information consumption-related indexes, basic geographic data, and other publicly available regional economic development related data, we conduct the exploration with the spatial Durbin model (SDM). The study indicates that (1) from the perspective of spatial analysis, the economic development level of all prefecture-level cities in Zhejiang, China has a significant positive spatial correlation; (2) in rural areas, the network infrastructure improvement, information industry clustering, etc. can accelerate enhancing information consumption and promote regional economic development; and (3) facilitate the development of rural information consumption is conducive to alleviate the imbalance of regional economic development. It is hoped that our study can help understand the current situation of information consumption in rural China and provide a theoretical reference for rural economic and social development.

## 1 Introduction

In recent years, the rapid development of information consumption led by e-commerce has provided a new impetus for the development of China’s regional economy. According to the report on "China Information Development Trend Report 2022 [1, 2], the revenue scale of China’s information consumption industry exceeded RMB 23.2 trillion in 2020, an increase of 10.1% year-on-year, with a growth rate of one percent compared to 2019. In order to promote the development of information consumption, many scholars have done a lot of research on the theory, behavior and demonstration of information consumption in China [3]. Most of the studies have shown that information consumption covers production consumption, living consumption, management consumption and other fields. With the rapid development of the new generation of information technology and information industry, various forms of information products are widely introduced into households [4]. Under the circumstances, information consumption gradually shows the role of driving economic development.

Rural information consumption is deemed as a new market for the development of the Internet. However, rural information consumption has received relatively little attention from the academic community for a long time. Compared to urban residents, rural residents’ information consumption in China is relatively low [5]. The spatial concentration and uneven development of information consumption in rural areas of China are more significant, and the gap between urban and rural areas is likely to expand if the government does not control it [6]. In March 2021, the Chinese government proposed a clear roadmap to coordinate development between rural and urban areas to promote the prosperity of the regional economy in the future [7]. Previous studies have put forward the theoretical framework of developing rural information consumption to promote regional economic development in China. Nevertheless, rare quantitative explorations have been reported on the linkage between them.

Therefore, in the study, we focus on quantitative analysis of the relationship between rural information consumption and regional economy development with a case study of Zhejiang, China. Contributions of this study are mainly in the following three aspects.

1. We quantitatively study the impact of information consumption on regional economic growth and social development in Zhejiang, China. Based on the existing research results, we constructed 0–1 neighborhood and economic-distance weight matrices, which consider the synergistic effect of various factors on information consumption agglomeration. Then, SDM was used to explore the influence of information consumption on regional economic growth in depth.
2. We explore the imbalance of regional economic development in Zhejiang, China, from a geographical point of view. With the basic geographic information data and publicly available economic & social development indexes, we use the Moran I approach to investigate the spatial agglomeration characteristics of municipal regional economies.
3. We study the relationship between information consumption in rural areas and regional economic development in Zhejiang, China. According to the dynamic characteristics of information consumption, publicly available data from 2011 to 2020 were collected, and SDM was used to explore the relationship between regional information consumption and economic & social development. In addition, the applicability of the model was verified using the Moran’s index, Lagrange multiplier LM test, LR test, and Wald test.

## 2 Literature review

### 2.1 Regional economic development influencing factors

The regional economies are generally considered as a part of national economy. They are usually formed through long-term socio-economic activities, such as historical, geographic, political, economic, or religious connections [8–10]. In these activities, some residential areas with frequent economic and other connections gradually form a distinctive economic region, while other connections gradually form their own distinctive economic zones. Based on the Perroux growth pole theory, scholars have proposed many theories, such as the cumulative causality theory [11], scholars have proposed many theories, such as the cumulative causality theory, the unbalanced growth theory [12], the inverted "U" theory [11], and Paul Krugman’s product life cycle theory [13] to explain the dynamic of regional economic development. Most existing studies suggest that regional development can be affected by many factors such as population aggregation and industrial agglomeration, development strategy, endowment of resources, and informatization and technological innovation ability [14, 15].

Information consumption is a kind of economic activity that directly or indirectly takes information products and information services as consumption objects. In the context of China’s vigorous development of digital economy, information consumption is considered to be an important driving force for regional economic and social development [16]. Because information consumption-related industries have created a large number of job opportunities, and information consumption has become an important force to promote industrial transformation and upgrading in China [17, 18]. The new economic geography theory successfully incorporates spatial factors into the research framework of the relationship between information industrial agglomeration and economic growth [13, 19]. Scholars argue from a micro perspective that knowledge or technology spillovers can cause economic agglomeration and thus promote economic development. Quantitative research from Romer and Lucas also indicates that technological innovation, human capital accumulation, and knowledge spillover have significant effects on regional economic growth [20, 21]. Coenen pointed out that the evolution of economic geography provides new clues to innovation and economic growth (IEG), which has the potential to improve analytical inputs to the IEG approach [22]. This approach improves our understanding of IEG dynamics in three different dimensions, while influencing policymaking for regional economic development [23]. In addition, spatial econometric-related studies indicate that regional economic growth processes and development spaces have a significant dependence [24, 25].

### 2.2 Relationship between rural information consumption and regional economic development

The development of rural information consumption is of great significance for alleviating the economic gap between urban and rural areas. The progress of science and technology and rapid development of the digital economy have significantly changed the traditional factor flow and resource allocation patterns between different regions [26]. The regions, of course, include rural areas. With China’s further urbanization, a metropolitan area economy, with cities as the center of development, has gradually formed. However, rural areas, as the outermost circles, have received relatively little attention over a long period. The concept of a metropolitan circle was first proposed by Japanese scholars as an urban system with at least one large city with strong radiation as the core to drive the surrounding cities to form a closer economic connection. Spatially, central cities and peripheral areas are formed in a circular structure [27, 28]. Based on the theory of metropolitan areas, Hu Meng constructed a four-level regional classification system with the scale of a metropolitan area in China and measured the gap between regions by using the three-level nested Thiel index decomposition method [29]. Their study showed that more attention should be paid to rural economic development. However, rural China’s reality is that a low knowledge level, remote location, and poor traffic environment result in a high investment costs in rural areas. Even if some policies based on the consideration of urban-rural balance are implemented, they are still "blossoming but not bearing fruit." Along with the improvement in e-commerce layout in China, rural e-commerce has been developing rapidly in recent years. This makes information consumption a crucial factor in promoting the coordinated development of urban and rural economies [30]. At present, the geopolitical isolation caused by the global coronavirus epidemic highlights the role of information consumption in regional economic development. Under these circumstances, the implementation of a spatial monetary policy is indispensable for achieving coordinated development of the regional economy [31].

How rural information consumption contributes to regional economic development has long been of interest to Chinese scholars. In early theoretical studies on the relationship between information consumption and rural regional economic growth, most researchers ignored spatial factors. By comparing information and traditional consumption and digging deeper into information consumption’s connotation, Ding argued that information consumption is different from general consumption, and its impact on the national economy is global, disruptive, and innovative [32]. He also believed that the way information consumption promotes national economic development was not simply by injecting technology into enterprises to improve their efficiency and reduce their costs, but deeply penetrating technology, reforming the production methods and organizational structures of traditional industries to achieve the transformation from "technology to economy."

From a microscopic perspective, a lot of research has focused on the diving force of rural information consumption to regional economic and social development. With the booming of e-commerce in China, it has become a typical representative of rural information consumption [33, 34]. The first three Taobao villages appeared in the Yangtze River Delta in 2009, and its number has increased by 705 times eight years later, spreading to 24 provinces and regions across China. According to AliResearch, by the end of August 2016, more than 840,000 jobs had been directly created by active online stores in Taobao villages [35]. Driven by rural e-commerce, the flow of goods between urban and rural areas has been opened up, and more capital flows to rural areas, which also drives the wave of the youth returning home to start businesses. Shirk John C and Shirk analyzed consumers’ information needs, cognitive processes, and behavioral barriers. They found that information consumption could be promoted by increasing information security and people’s educational levels [36, 37]. Liu and Zhang used the Thiel index, Gini index, geographical weighted regression, and other analytical methods to study the relationship between information consumption in different regions and information consumption to promote regional economic development. Their studies reveal that rural information consumption is a dynamic process, and its impact on the development of the regional economy grows almost exponentially with the progress of science and technology [5, 6].

In general, most existing studies explore the spatial distribution pattern of information consumption for regional economic development at the national or provincial level in China. However, relatively few studies have focused on the scale of cities, county-level cities, and counties, and research on rural information consumption is scarce. The SDM model was proposed by LeSage and Pace using spatially lagged explanatory variables, which can effectively exclude the error term of spatial autocorrelation and explain the dynamic properties of information consumption development [38]. Therefore, we selected this model to explore the dynamic evolution and rapid development of information consumption in the rural areas of Zhejiang, China. It is hoped that the conclusions of this research can provide valuable suggestions for regional development decision-making.

## 3 Study area and data sources

### 3.1 Study area

**Fig 1:**
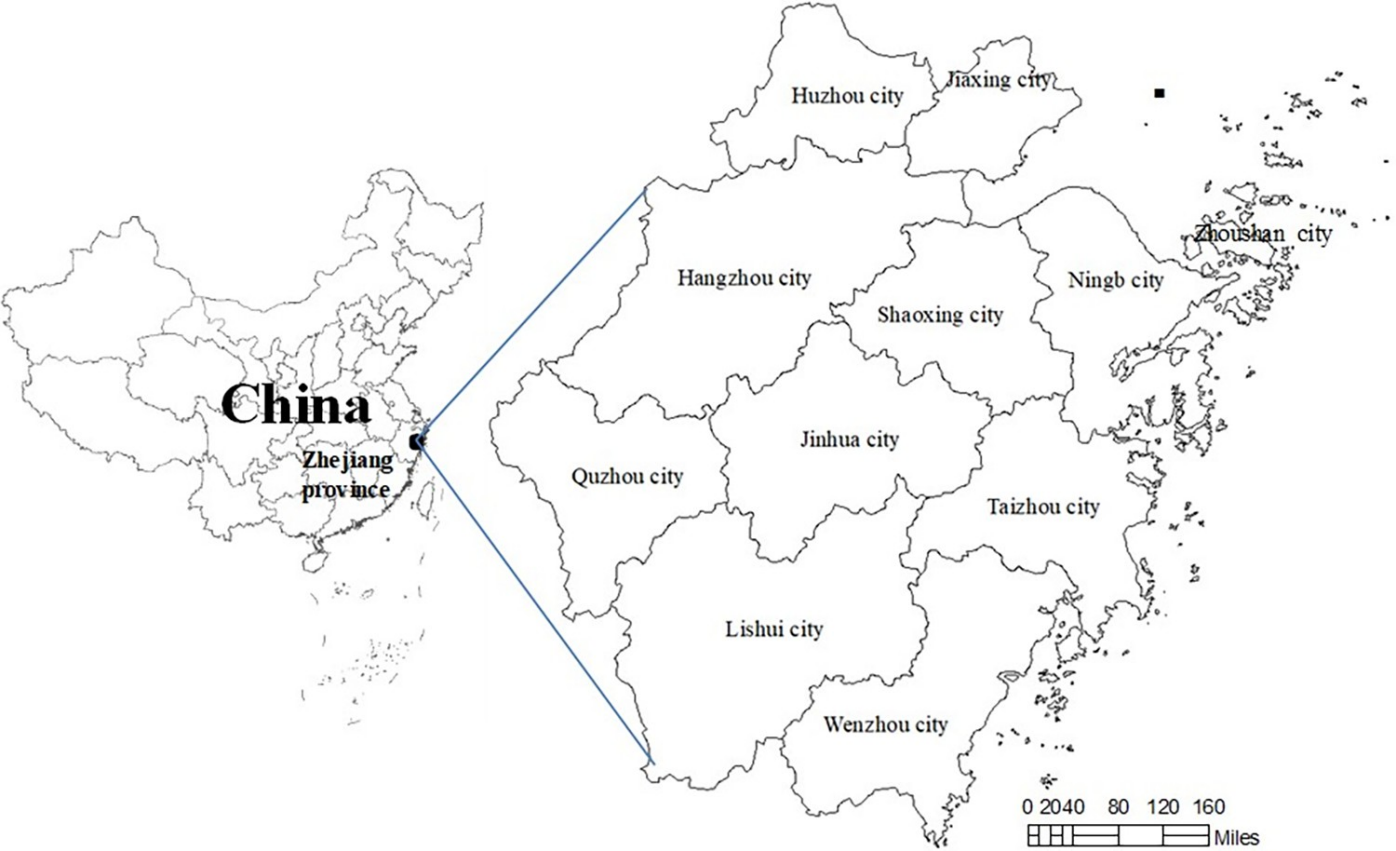
The geographical location of Zhejiang province.

Zhejiang, China, is located in the east coast of China and has developed economy. The province is mountainous and has complex terrain, but it has superior natural conditions. Zhejiang, China has 11 cities (including two sub-provincial cities) area under administration and the location of the province is shown in Fig 1. In cities under the jurisdiction of Zhejiang, China, Hangzhou, Ningbo, Shaoxing, and Wenzhou were the four economic pillars [39].

In 2019, Zhejiang, China was selected as the national pilot zone for digital economic innovation and development. Two years later, the province was selected as the first common prosperity demonstration zone in China [40]. In 2021, Zhejiang, China decided to implement the digital economy "No. 1 project" deeply, which accelerated the construction of national digital economy innovation and vigorously cultivated digital industry clusters and made industrial digitalization index of the province rank the first in China.

Zhejiang, China is deemed as the earliest birthplace of e-commerce in China, and the information economy in the province has recently developed rapidly. The giant global e-commerce Alibaba Group is located in the capital city of Zhejiang, China. The province’s economic aggregate has long been among the top provincial levels in China, and the urban-rural economic development gap in the province is small [41]. All these factors make it conducive to studying the synergistic and promoting effects of rural information consumption on regional economic development. Therefore, this study selected Zhejiang, China, as a case study to analyze the spatial pattern of rural information consumption and its intrinsic mechanism for promoting regional economic development.

### 3.2 Data sources

The dataset applied in this study fall into three categories: (1) rural information consumption–related data. These data were collected from the Department of Commerce of Zhejiang Province (http://www.zcom.gov.cn), the Zhejiang Provincial Bureau of Statistics (http://tjj.zj.gov.cn), and the statistical bureaus of cities administrated by Zhejiang, China. (2) Regional economic and social development related data, such as health care, education, recreation, GDP, and transportation and communication. These data were extracted from the statistical yearbooks of 11 cities administered by Zhejiang, China from 2012 to 2021, and (3) basic geographical data of Zhejiang, China. The data were collected from the National Science and Technology Infrastructure website (http://www.bjshrimp.cn/cn/introduction). After simple splitting, merging, and other operations, the corresponding data can be used for the analytical investigation.

## 4 Research methods

**Fig 2:**
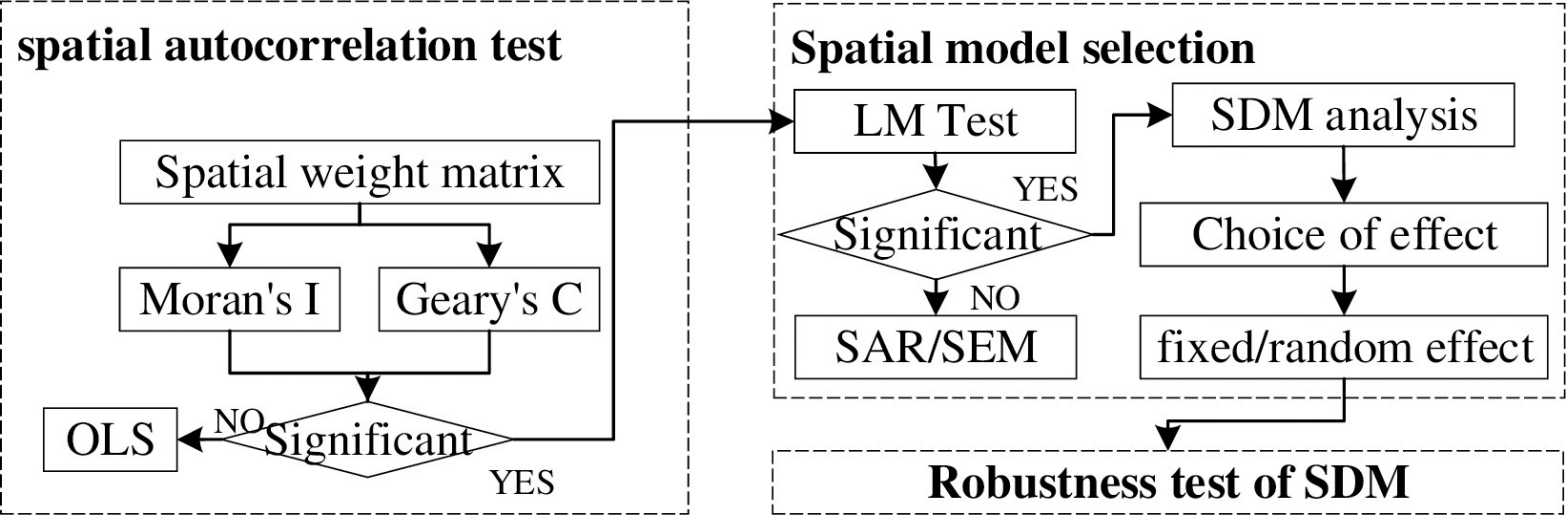
Workflow of the research method.

The whole approach of the study falls into three steps: (1) spatial autocorrelation test: check whether the collected data indictors are suitable for SDM analysis, (2) spatial model selection: determine the appropriate model for SDM analysis with Lagrange multiplier test and effect selection evaluation, and (3) robustness test of SDM: examine whether the selected model is robust with Wald and LR tests. The general workflow of the research method is shown in Fig 2.

### 4.1 SDM analysis

#### 4.1.1 Spatial autocorrelation test

The spatial weight matrix is an important tool for measuring the spatial effects in different areas. This is the core content of spatial metrology and is often used to represent the spatial dependence structure. Therefore, this study constructs two kinds of spatial weight matrices according to the characteristics of the research content to compare and analyze the dependence degree and spillover effect between the information consumption level of rural residents and regional economic growth.

*Spatial adjacency weight matrix* (*W*_1_). In this study, Rook adjacency method [42] is adopted to construct the spatial adjacency weight matrix. The specific idea is that if two adjacent spatial units have a common edge, the value is assigned a value of 1; otherwise, the value is 0. ***W***_**1**_ can be expressed as follows:

$$w_{ij}=\left\{ \begin{array}{l} 1\;bound(i)\cap bound(j)\neq \emptyset \\ 0\;bound(i)\cap bound(j)=\emptyset \end{array}\right.\;i\neq j$$ (1)

Where *bound*(*i*) is the boundary of the spot *i*.

*Economic distance weight matrix (W*_2_*)*. It considers the reciprocal of the difference between the per capita income levels of the two cities as the economic distance parameter. In this study, the average GDP per capita of each prefecture-level city in Zhejiang, China from 2011 to 2020 was selected to represent the per capita income level. The specific construction concept of ***W***_**2**_ is as follows.

$$w_{ij}=\left\{ \begin{array}{c} \frac{1}{\left|\bar{X_{I}}-\bar{X_{j}}\right|}i\neq j\\ \;0\;i=j \end{array}\right.$$ (2)

where $\bar{X_{I}}$ and $\bar{X_{J}}$ represent the average GDP per capita of Cities I and *J*, respectively.

*Spatial autocorrelation analysis*. The purpose of the spatial autocorrelation test is to verify whether similar variables exist in neighboring regions to form regional spatial agglomeration. If similar attribute indicators accumulate, it is called a positive spatial correlation; otherwise, it is called a negative correlation. If the test values are randomly distributed, then the spatial effect between them does not exist or is weak. In recent years, Moran’s I, Geary’s C, and the Getis-ord G index have been proposed to measure the degree of spatial autocorrelation status. Based on the summary and analysis of the existing research results, Moran’s I index approach was selected to test and verify the spatial autocorrelation between rural information consumption and regional economic development. The Moran’s I calculation method is shown in Eq (3) below.


$$Moran’s\;I=\frac{\sum_{i=1}^{n}\sum_{j=1}^{n}w_{i,j}(x_{i}-\bar{x)}(x_{j}-\bar{x)}}{{s_{o}}^{2}\sum_{i=1}^{n}\sum_{j=1}^{n}w_{i,j}}$$ (3)

$${s_{o}}^{2}=\frac{\sum_{i=1}^{n}{(x_{i}-\bar{x)}}^{2}}{n},\bar{x}=\frac{\sum_{i=1}^{n}x_{i}}{n}$$ (4)

The calculation formula of Geary’s C is as follows.


$$Geary’s\;C=\frac{\sum_{i=1}^{n}\sum_{j=1}^{n}w_{ij}{\left(x_{i}-x_{j}\right)}^{2}}{2ns^{2}\sum_{i=1}^{n}\sum_{j=1}^{n}w_{ij}}$$ (5)

$$s^{2}=\frac{\sum_{i=1}^{n}{(x_{i}-\bar{x)}}^{2}}{n}$$ (6)

In Eqs (3) to (6), *w*_*i*,*j*_ represents the spatial weight between elements *i* and *j*, *x*_*i*_ is the observed value of the *i*th element, and *n* is the total number of elements. According to the equation, the value of Moran’s I is generally between -1 and 1; If Moran’s I value is greater than 0, it implies there is a positive correlation, and the larger the value of Moran’s I, the higher the spatial correlation; If Moran’s I value is smaller than 0, it implies there is a negative correlation, and the smaller the value of Moran’s I, the higher the spatial correlation; If Moran’s I value is equal to 0, it implies the variables does not have a correlation. According to Eq (5), the value of Geary’s C is between 0 and 2. If the Geary’s C value is greater than 1, it implies a positive correlation, and the higher the value of Geary’s C, the higher the spatial correlation; If the Geary’s C value is smaller than 1, it implies a negative correlation, and the smaller the value of Geary’s C, the lower the spatial correlation; If the Geary’s C value equals to 1, it implies no correlation between variables.

#### 4.1.2 Spatial model selection

In the empirical study of spatial econometrics, it is important to select a relatively appropriate spatial econometrics model. In recent years, spatial autoregressive model (SAR), spatial error model (SEM), and SDM have been frequently used in the literature on spatial problems. Using different models for different data-generation processes will lead to different unbiased and effective estimates. First, if the model corresponds to the data generation process, the estimation is unbiased and efficient. Second, the SDM contains the control variables of the explanatory variables. If these rules are ignored, and other models are used, the estimation will be biased. Finally, ignoring the spatial lag of the dependent variable may result in biased results. Therefore, the following validation was required to determine the appropriate model:

(1) Lagrange multiplier test (LM test)

**Fig 3:**
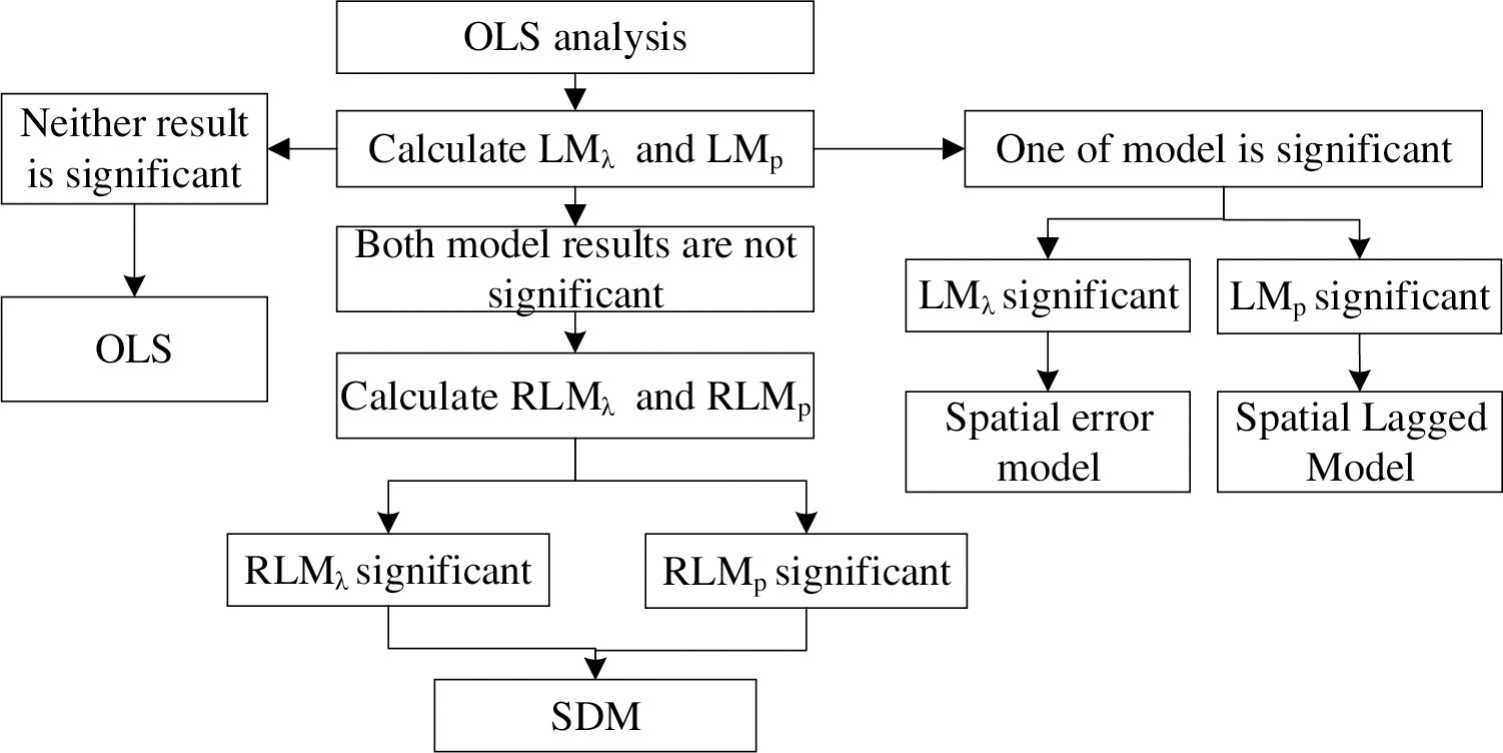
Model selection process.

Based on the concept of the Lagrange multiplier test, the following flow chart of spatial model selection is presented in this paper, as shown in Fig 3.

In Fig 3, *LM*_*λ*_ and *LM*_*ρ*_ represent the classical Lagrange multiplier test of SEM and SAR, respectively. *RLM*_*λ*_ and *RLM*_*ρ*_ represent the Lagrange multiplier tests of SEM and SAR, respectively. The LM test [43] is based on the estimation results of least-squares residuals and spatial weight matrices involved, and the maximization of the log-likelihood function under constrained conditions should be considered. Therefore, before the LM test, a least squares regression (OLS) was conducted to obtain the residual. The specific formula is as follows:

$$y=\alpha l_{N}+X\beta +\varepsilon$$ (7)

where *y* is an N*1 order matrix composed of observed values corresponding to each spatial unit of the explained variable in the sample. *l*_*N*_ is a unit N × 1 cell column vector, *α* is a constant, *X* is an exogenous explanatory variable, *ε* is the random error vector, and β is a parameter matrix.

The LM test formula is as follows:

$${LM}_{\lambda }=\frac{{\left(ne^{\prime }We/ee^{\prime }\right)}^{2}}{tr((W+W^{\prime })W)}∼x^{2}(1)$$ (8)

The relationship between *LM*_*λ*_ and *I*_*Moran*_ (the statistics of *Moran’s I*) is as follows:

$${LM}_{\lambda }=\frac{n^{2}{I^{2}}_{Moran}}{tr((W+W^{\prime })W)}$$ (9)

$${LM}_{\rho }=\frac{{\left(ne^{\prime }Wy/e^{\prime }e\right)}^{2}}{tr((W+W^{\prime })W)+n{\left(WX\beta \right)}^{\prime }+(I_{n}-X{\left(X^{\prime }X\right)}^{-1}X^{\prime })(WX\beta )/e^{\prime }e}∼x^{2}$$ (10)

$${RLM}_{\lambda }=\frac{{\tilde{d}}_{\lambda }-TD^{-1}{\tilde{d}}_{\rho }}{T(1-TD)}∼x^{2}(1)$$ (11)

$${RLM}_{\rho }=\frac{{\left({\tilde{d}}_{\lambda }-{\tilde{d}}_{\rho }\right)}^{2}}{D-T}∼x^{2}(1)$$ (12)

$$Where\;{\tilde{d}}_{\lambda }=\frac{e^{\prime }We}{n^{-1}e^{\prime }e},{\tilde{d}}_{\rho }=\frac{e^{\prime }Wy}{n^{-1}e^{\prime }e},T=tr((W+W^{\prime })W)$$ (13)

$$D=T+\frac{{\left(WX\tilde{\beta }\right)}^{\prime }(I_{n}-X{\left(X^{\prime }X\right)}^{\prime }(WX\tilde{\beta })}{n^{-1}e^{\prime }e}$$ (14)

where *e* represents the least-squares residual, *M* represents the number of constraints, *T* represents the sample size, and $\tilde{\beta }$ represents the coefficient estimate estimated by the least-squares method.

(2) Choice of effect

Effect selection was divided into two parts: fixed/random effect selection and individual/time/dual fixed model selection.

In fixed/random effects selection, the Hausman test is a commonly used method that assumes that individual effects are independent of the exogenous variables. Under the assumption of a normal distribution, the estimated values of the random-effects space panel model obtained using the maximum likelihood estimation $\hat{\theta_{r}}$ are consistent and asymptotically efficient. However, if the individual effects are correlated with exogenous variables, then the estimates are inconsistent and the fixed effects model estimate $\hat{\theta_{f}}$ is consistent.

In the selection of volume/time/dual fixed models, this study used the likelihood ratio test (LR test) to evaluate the three models, and four factors, goodness-of-fit, *sigma*^2^, log-likelihood function value, and *ρ*, were considered comprehensively to select the appropriate model for the next calculation. The specific formula for the LR test is as follows:

$${LR}_{\rho }=n[Ln(\sigma_{0}^{2})-Ln(\sigma_{1}^{2})]+2Ln|I_{n}-\rho W|∼x^{2}(1)$$ (15)

where $\sigma_{0}^{2}$ is the estimated *σ*^2^ value when *p* = 0, and $\sigma_{1}^{2}$ is the estimated *σ*^2^ when *p*≠0.

### 4.2 Robustness test of SDM

The robustness test plays a very important role in examining the explanatory ability of the evaluation method and the selected indicators. Specifically, it examines whether they maintain a consistent and stable interpretation of the evaluation results when certain parameters change. In this study, Wald and LR tests were selected to judge whether the selected model is robust.

In the robustness test, the null hypothesis of the maximum likelihood test and Wald test is *H*_0_ while *p* = 0, and the alternative hypothesis is *H*_1_ while *p* = 1. The specific formula for the Wald test is as follows:

$$WT_{\rho }={{(\rho }^{2}(tr(B^{2})+tr(B^{\prime }B)-\frac{2}{n}(tr(B))}^{2}+\sigma^{-2}\beta^{\prime }X^{\prime }B^{\prime }(I_{n}-X{\left(X^{\prime }X\right)}^{-1}X^{\prime })BX\beta )∼x^{2}(1)$$ (16)

Where *β*, *σ*^2^ and *ρ* are the maximum likelihood estimates.

If *prob*>*chi*2 in the SDM analysis result, it indicates that the probability of an incorrect analysis conclusion is less than 1%, and the selected model is robust. Otherwise, an appropriate model would be required.

## 5 Empirical analysis

According to existing research, many factors affect regional economic growth. With reference to the practice in the literature, this research mainly selected rural residents’ consumption in each prefecture-level city of Zhejiang, China, as the dependent variable, and adopted rural disposable income, rural population size, etc. as independent variables to conduct the SDM analysis. The variables fall into three categories, and a detailed explanation is provided in Table 1.

**Table 1:** Variables description.

Variable Name	Variable Meaning	Average value (Yuan)	Standard deviation	Minimum value (Yuan)	Maximum value (Yuan)
Regional economic development level (v1)	GDP per capita in each city	80287.66	27751.96	36508	152465
Information consumption level of rural residents (v2)	Rural residents transportation and communication expenditures + recreation and entertainment expenditures + health care expenditures	5076.85	1762.07	1669.00	9141.00
rural per capita disposable income (v3)	The sum of consumption and savings	23840.89	7846.18	7809.00	39801.00
Urbanization level (v4)	Ratio of urban population to total population in each municipality	0.64	0.07	0.45	0.83
Information industry agglomeration status (v5)	Added value of Information transmission, software and information technology services	220.63	534.63	2.44	3170.21
Online retail sales,(v6)	Total volume of online retail imports and exports in each city	934.49	1401.48	3.20	8992.20
Internet user scale (v7)	Number of mobile internet users	737.57	461.46	123.16	1913.91

Data source: Statistical Yearbook of cities in Zhejiang, China (http://tjj.zj.gov.cn/)

Explained variable: regional economic development level expressed by GDP per capita of all regions and cities [44].

Core explanatory variable: Rural residents’ information consumption level, which mainly consists of transportation and communication, health care, and education and recreation of rural residents [6, 14].

Control variables: Rural per capita disposable income, urbanization level, information industry agglomeration status, online retail sales, and Internet user scale.

### 5.1 Spatial autocorrelation test

The widely used spatial autocorrelation testing methods include Moran’s I, Geary’s C, Getis’s G, and the semi-variance function. Based on current research findings, this study mainly adopts Moran’s I to determine whether there is spatial dependence on the economic development level in Zhejiang, China. We set the economic distance weight matrix value according to the inverse of the average income gap between the cities in the province. The smaller the income gap between the two cities, the greater the weight, and vice versa.

With the help of Stata (https://www.stata.com/products/windows/) software, we explored the spatial pattern of the economic development level of Zhejiang, China, and the results are shown in Table 2. Spatial statistical analysis shows that the Moran I and Geary’s C values of the economic development level of Zhejiang, China are significantly positive for both w_1_ and w_2_. This indicates that the economic development aggregation situation is significant, and uneven economic and social development is obvious in the province. However, the spatial dependence of the economic development level in Zhejiang, China, was not significant in 2020. This may have been caused by the implementation of regional isolation in various parts of the province owing to the sudden outbreak of COVID-19 in 2020.

**Table 2:** Spatial autocorrelation test.

	Regional economic development level							
	*Moran’s I*				*Geary’s C*			
Spatial weight matrix	*W*_1_		*W*_2_		*W*_1_		*W*_2_	
Variables	*I*	p-value*	*I*	p-value*	*I*	p-value*	*I*	p-value*
2011	0.425	0.011	0.227	0.076	0.533	0.016	0.597	0.021
2012	0.422	0.012	0.190	0.116	0.510	0.012	0.631	0.034
2013	0.465	0.005	0.206	0.092	0.552	0.022	0.596	0.022
2014	0.386	0.017	0.211	0.088	0.576	0.030	0.606	0.025
2015	0.413	0.012	0.238	0.065	0.572	0.028	0.579	0.016
2016	0.407	0.013	0.249	0.056	0.570	0.028	0.566	0.013
2017	0.412	0.012	0.241	0.060	0.596	0.039	0.570	0.015
2018	0.429	0.010	0.245	0.058	0.579	0.031	0.573	0.015
2019	0.443	0.008	0.256	0.050	0.578	0.031	0.560	0.012
2020	0.485	0.005	0.295	0.032	0.512	0.012	0.546	0.009

Note: w_1_ is the spatial adjacency weight matrix, w_2_ is the economic distance weight matrix, p-value* is the significant value.

### 5.2 SDM applicability test

#### 5.2.1 LM test

As shown in Table 2, the regression coefficients of the three independent variables of disposable income per capita, information industry agglomeration, and Internet user size are positive and significant. However, a Lagrange multiplier test is required to identify whether the regression results are reliable. In this study, we perform statistical tests on the residuals obtained from the regressions using a spatial weight distance matrix and an economic weight distance matrix. As can be seen in Table 3, both ***W***_**1**_ and ***W***_**2**_ LM tests are significant, indicating that the original hypothesis of "no spatial lag and spatial error" is rejected.

**Table 3:** Model selection test.

Variables	OLS	LM test					
		*W*_1_			*W*_2_		
v1	Coef	Test	Statistic	p-value	Test	Statistic	p-value
v2	0.937***(10.38)	**Spatial error**			**Spatial error**		
v3	0.058***(3.68)	Moran’s I	1.076	0.282	Moran’s I	2.501	0.016
v4	-0.117(-0.34)	Lagrange multiplier	5.06	0.024	Lagrange multiplier	14.699	0.031
v5	-0.064***(-4.91)	Robust Lagrange multiplier	5.019	0.025	Robust Lagrange multiplier	4.153	0.042
v6	-0.213***(-6.98)	**Spatial lag:**			**Spatial lag:**		
v7	0.327***(6.04)	Lagrange multiplier	8.073	0.023	Lagrange multiplier	14.475	0.002
_cons	3.063***(3.83)	Robust Lagrange multiplier	5.032	0.018	Robust Lagrange multiplier	6.928	0.004

#### 5.2.2 Simple effect test

To verify whether the SDM selected fixed effects or random effects, we analyzed the data using the Hausman test, and the results are shown in Table 4. As shown in the table, *prob*>*chi2* with both the spatial adjacency matrix and spatial economic weight matrix. Therefore, we can draw a conclusion that the null hypothesis is rejected, and the fixed effects are dependable for further analysis.

**Table 4:** Hausman test result.

Variables	*W*_1_			*W*_2_		
	Time fixed effect	Individual fixed effect	Dual fixed effect	Time fixed effect	Individual fixed effect	Dual fixed effect
*r* ^2^	0.896	0.88	0.294	0.630	0.817	0.309
Log-likelihood	86.1491	161.3181	173.6663	99.9646	156.0448	274.8575
*ρ*	0.159***	0.108***	-0.003	-0.294***	0.293**	-0.312**
	(-3.08)	(-3.16)	(-0.03)	(-3.46)	(-2.48)	(-2.00)
*sigma* ^2^	0.012***	0.003***	0.002***	0.009*	0.003*	0.002***
	(7.23)	(7.35)	(7.42)	(7.13)	(7.34)	(7.29)
N	110	110	110	110	110	110
**Hausman test**						
	2.9e+10 ***(p = 0.000)			3.5e+10 ***(p = 0.000)		

Note: Coefficient estimates are t-statistics in parentheses; p-values in parentheses in Hausman test

*** represents p<0.01

** represents p<0.05, and

* represents p<0.1

According to the likelihood ratio test with w_1_, we find: (1) in terms of goodness of fit, the time fixed effect is the best, the dual fixed effect is the second, and the individual fixed effect is the worst; (2) in terms of *sigma*^2^, all three modes passed the 1% significance test; (3) in terms of log-likelihood values, the dual fixed effect is the best, followed by individual fixed effect and time fixed effect; and (4) in terms of *p* value, the individual fixed effect and dual fixed effect are significant at 1%, respectively, while the time fixed effect is not significant.

Similarly, the likelihood ratio test with w_2_, we find the following: (1) in terms of goodness of fit, the individual fixed effect is the best, the time fixed effect is the second, and the dual fixed effect is the worst; (2) in terms of *sigma*^2^, all three modes passed the 1% significance test; (3) in terms of log-likelihood values, the dual fixed effect is the best, followed by individual fixed effect and time fixed effect; and (4) in terms of *p* value, the individual fixed effect and dual fixed effect are significant at the 5% level, while the time fixed effect is not significant.

Based on the likelihood ratio test with w_1_ and w_2_, we conclude that SDM with individual fixed effects is reliable for further analysis.

#### 5.2.3 Validation of spatial model estimation

To further determine the applicability of SDM, the Wald test was selected to verify whether the SDM might be transformed into SAR or SEM. To ensure the regression structure’s accuracy, this study adopted the maximum likelihood estimation method for the analysis and evaluation. Detailed regression results are presented in Table 5. Through regression estimation, we find the Wald test rejected the null hypothesis and *prob*>*chi2*, with w_1_ and w_2_, respectively. This verifies that SDM cannot be transformed into SAR and SEM models and further indicates the investigation results of SDM is credible.

**Table 5:** Spatial model estimation.

Variables	*W*_1_		*W*_2_	
	SAR	SEM	SAR	SEM
LR test	LR chi2(6) = 50.09	LR chi2(6) = 73.03	LR chi2(6) = 70.37	LR chi2(6) = 117.29
	Prob>chi2 = 0.0000	Prob>chi2 = 0.0000	Prob>chi2 = 0.0000	Prob>chi2 = 0.0000
Wald test	chi2(6) = 39.80	chi2(6) = 59.86	chi2(6) = 132.31	chi2(6) = 235.21
	Prob>chi2 = 0.0384	Prob>chi2 = 0.0000	Prob>chi2 = 0.0000	Prob>chi2 = 0.0000

Note: t-statistics in coefficient estimates in parentheses

### 5.3 SDM analysis

The SDM analysis results are presented in Table 6, where *Main* represents the significant effect of the explanatory variable on the explained variable, *and Wx* represents the spatial spillover effect of the explanatory variable on the explained variable. As shown in Table 6, the model fits well with high confidence, measured by *r*^2^ and *sigma*^2^; the spatial autocorrelation coefficient passes the 1% significance test with both ***W***_**1**_ and ***W***_**2**_. This indicate an obvious spatial dependence of the development of the rural economy in Zhejiang, China. The negative coefficient, according to the echo effect theory [45], may reveal that rural economic development in Zhejiang, China, is still in a crude economic growth mode, which mainly relies on increasing the input of production factors, that is, increasing investment, expanding plants, and increasing labor input, to increase production. In essence, this economic growth method is to increase output by increasing resource inputs. This will inevitably affect the economic development of its surrounding areas, cause gratuitous waste, increase carbon emissions, and other problems. This economic growth is not sustainable and is inconsistent with China’s current green production concept.

**Table 6:** Estimation results of SDM.

Variable	*W*_1_		*W*_2_	
	*Main*	*Wx*	*Main*	*Wx*
v2	0.0232**(0.125)	-0.149**(-2.47)	0.082***(0.307)	0.077***(0.64)
v3	0.236***(5.03)	-0.159*(-1.86)	0.235***(3.14)	-0.301***(-3.12)
v4	0.362(0.51)	-0.439(-0.80)	-0.664**(-2.49)	0.500(4.76)
v5	0.069***(7.00)	-0.031(-1.19)	0.065***(5.24)	0.146***(5.36)
v6	0.112***(3.98)	-0.201***(-2.86)	0.187***(6.91)	0.322***(4.79)
v7	0.002(1.02)	-0.032(-0.15)	0.066(1.06)	0.733***(5.12)
*ρ*	-0.359***(-3.08)		*ρ*	-0.451*** (-1.54)
*sigma* ^2^	0.002***(5.23)		*sigma* ^2^	0.00126*** (5.42)
*r* ^2^	0.896		*r* ^2^	0.831

Note: The z values are in parentheses

*** represents p<0.01

** represents p<0.05, and

* represents p<0.1

Regional economic and social development depends on each other. Rural information consumption cannot be simply used as an explanation for regional economic development, and the spatial spillover effect should be taken into account. Therefore, the direct and indirect effects obtained from the decomposition of the total spatial effects of rural information consumption are analyzed to gain a more comprehensive understanding of the direct and indirect effects of rural information consumption on regional economic development. The results are listed in Table 7, where LR-Direct represents the influence of the independent variables of a certain region on the dependent variable, including feedback effects. Numerically, direct effect equals to the feedback effect plus coefficients of SDM; LR_Indirect effect is generally used to measure the impact of an explanatory variable in a "neighboring" region on the explained variable in the region; LR_Total represents the average influence of the change in an explanatory variable in a certain region on the explained variables in all the "neighboring" regions. Specific conclusions can be drawn from Table 7 as follows.

**Table 7:** Direct, indirect and total effects of SDM.

Variable	*W*_1_			*W*_2_		
	LR-Direct	LR-Indirect	LR-Total	LR-Direct	LR-Indirect	LR-Total
v2	0.302**(2.65)	-0.842***(-3.05)	-1.144***(-3.36)	0.780***(12.03)	0.299(1.56)	1.079***(5.74)
v3	0.262**(6.04)	-0.351***(-2.75)	0.612*(3.89)	0.208***(5.33)	-0.149**(-2.15)	0.357***(5.92)
v4	0.390(1.21)	-0.745(-0.88)	-1.135(-1.08)	-0.617**(-1.98)	0.118(0.21)	0.734*(1.71)
v5	0.068**(5.52)	-0.011(-0.26)	0.057(1.07)	0.050***(5.47)	0.091***(4.90)	0.141***(7.52)
v6	0.141**(4.86)	-0.358***(-3.19)	0.499***(3.91)	0.158***(6.42)	0.185***(3.04)	0.342***(5.39)
v7	0.001(0.01)	-0.030(-0.10)	-0.031(-0.08)	0.024(0.34)	-0.568***(-4.24)	0.544***(2.66)

Note: The z values are in parentheses

*** represents p<0.01

** represents p<0.05, and

* represents p<0.1

The first is the direct effect of SDM. With the two different weight matrices ***W***_**1**_ and ***W***_**2**_, the impact coefficients of rural residents’ information consumption level on economic growth within the region are 0.302 and 0.780, respectively. This indicates that the information consumption level of rural residents has a positive promoting effect on the development of regional economic. As shown in Table 3, the coefficient of the information consumption of rural residents on the regional economic development level estimated by OLS is 0.937. This indicates that the role of rural residents’ information consumption in regional economic development will be underestimated without considering the spatial spillover effect. In addition, the effect of rural residents’ information consumption on economic growth with ***W***_**1**_ is greater than that of ***W***_**2**_, indicating that after considering economic factors, the promoting effect of rural residents’ information consumption level is weakened, which can also be explained by Murdal’s "echo effect" theory [45]. With the deepening of urbanization in China, industrial agglomeration has mainly occurred in urban areas. The resulting economies of scale will attract talent and technologies from surrounding backward areas to cities, inhibit the economic development of backward rural areas, and have a negative spillover effect. In addition, the impact of the information industry agglomeration and online retail sales is significantly positive for both ***W***_**1**_ and ***W***_**2**_. They are closely related to rural residents’ information consumption, which also proves that improving the level of information consumption is helpful for regional economic development and has positive significance for regional economy’s coordinated development.

Second, SDM had an indirect effect. Rural residents’ information consumption is negative for ***W***_**1**_ and passes the 5% significance level test. Meanwhile, the spillover effect of rural information consumption is positive, but not significant, with ***W***_**2**_ when economic factors are added. This indicates that the inhibitory effect of rural residents’ information consumption on economic growth in other regions is mitigated when economic factors are added. It can be explained by Muirda’s "diffusion effect" [45], which shows that the economic interaction between developed regions and neighboring backward regions can promote the spillover of technological knowledge, and the demonstration effect and driving effect of economic growth in developed regions will promote the economic development of their neighbors. That is, the increase in the information consumption level of rural residents in "adjacent" areas will produce an "echo effect" on local economic development.

Third, direct and indirect effects of the control variables. Quantitative analysis shows that the direct effect of rural residents’ disposable income on economic growth is significantly positive for *W*_1_ and *W*_2_, while the indirect effect is negative under the same conditions. The disposable income of rural residents comprises the funds used for consumption or savings. Consumption improves regional economic vitality, which is of great significance for the development of the regional economy. The analysis results in Table 7 show that the disposable income of rural residents makes it difficult to promote the development of the regional economy. We believe that the reasons might be: (1) although the consumption level of rural residents in Zhejiang, China is higher than the national average, the overall level is still low, and much lower than that of urban residents, and (2) the polarized distribution pattern of urban and rural areas in China objectively results in a long distance between cities and rural areas, while most of the major products are concentrated in cities. Despite the continuous development of e-commerce economy in recent years, rural residents have low enthusiasm for consumption because of the "last kilometer of logistics" and their low awareness of e-commerce. The direct effects of information industry agglomeration and online retail sales are significantly positive for *W*_1_ and *W*_2_. Information industry and online consumption are the result of "digital economy,” indicating that digital economy can effectively promote the development of a regional economy too.

### 5.4 Contribution of rural information consumption to economic development

In recent years, information consumption with e-commerce as the main force is accelerating to cover the rural areas and reshaping the consumption pattern of China’s rural society. The exchange of urban and rural elements caused by information flow, logistics and capital flow based on information consumption is the direct driving force for the reconstruction of rural regional system. As can be seen from the above Table 6, no matter in the spatial adjacency or economic distance weight matrix, the whole (Main) rural residents’ information consumption plays a significant role in promoting regional economic development. Although the lag factor is not included in the regression analysis, the coefficient of the explanatory variable may not directly reflect the influence of the explanatory variable on the explained variable, but the estimated coefficient of the explanatory variable can also reflect the influence of rural information consumption on regional economic growth to some extent. Considering the spatial spillover effect (Wx), the effects of information consumption on regional economic growth are all significant and positive, which indicates that rural residents’ information consumption has a strong "neighbor effect".

With the acceleration of China’s digitalization process, information consumption will further change the consumption habits of rural residents and exert a spatial spillover impact on surrounding people or regions, thus expanding the impact of information consumption on regional economy. Therefore, we believe that information consumption of rural residents will play an increasingly important role in regional economic development in the future.

## 6 Conclusions and future work

From the perspective of China’s national development strategy, the importance of rural areas is being widely concerned. However, insufficient attention has been paid on the important role of rural information consumption in promoting regional economic development. This paper has investigated the significance of rural information consumption with a case study of Zhejiang, China. SDM analysis with the basic geographic information data and panel data from 2011 to 2020 indicate regional economic development imbalance of the province is obvious. And the development of rural economy in Zhejiang, China has significant spatial dependence. Correlation analysis shows that the information consumption of rural residents in Zhejiang, China can promote the level of regional economic development to a certain extent and effectively improve the province’s uneven spatial distribution pattern. To accelerate the development level of the regional economy, more effort should be paid on the improvement of network infrastructure and the information industry agglomeration in rural areas.

Owing to the availability of data, consistency of statistical indicators, and other limitations, we collected publicly available panel data from Zhejiang, China, to conduct a prefecture-level city investigation. In the future, we will focus on gathering statistics for consecutive years to imply a finer scale study and make comparative studies from other provinces in China to obtain more specific and valuable findings.
